# Decreased biventricular longitudinal strain in patients with systemic sclerosis is mainly caused by pulmonary hypertension and not by systemic sclerosis *per se*

**DOI:** 10.1111/cpf.12561

**Published:** 2019-01-16

**Authors:** Anthony Lindholm, Roger Hesselstrand, Göran Rådegran, Håkan Arheden, Ellen Ostenfeld

**Affiliations:** ^1^ Department of Clinical Sciences Lund Clinical Physiology Skåne University Hospital Lund University Lund Sweden; ^2^ Department of Clinical Sciences Lund Rheumatology Skåne University Hospital Lund University Lund Sweden; ^3^ Department of Clinical Sciences Lund Cardiology Skåne University Hospital Lund University Lund Sweden

**Keywords:** cardiac magnetic resonance imaging, feature tracking, left ventricle, peak global longitudinal strain, pulmonary arterial hypertension, right ventricle

## Abstract

**Purpose:**

Patients with pulmonary arterial hypertension (PAH) due to systemic sclerosis (SSc) have high mortality. Left ventricular (LV) peak global longitudinal strain (GLS) is decreased in SSc. It is unknown whether low GLS is due to SSc or PAH. Therefore, our primary aim was to evaluate both LV and right ventricular free wall GLS (RVFW GLS) in SSc, with and without PAH, using cardiac magnetic resonance with feature tracking. Secondary aim was to relate GLS to invasive mean pulmonary arterial pressure (mPAP) and pulmonary vascular resistance (PVR).

**Methods:**

Thirty‐eight patients with SSc, 19 patients with SSc‐PAH and 19 healthy controls for comparison, were included. Endocardial and epicardial borders were delineated in cine images (short‐axis stack and three long‐axis views) for volumetric and strain calculations.

**Results:**

Systemic sclerosis‐PAH had lower LV and RVFW GLS than SSc (LV:* P* = 0·01, RV:* P*<0·001) and controls (LV:* P* = 0·02; RV:* P*<0·001), with no difference between SSc and controls. LV strain correlated with mPAP (*R* = 0·42, *P* = 0·03) and PVR (*R* = 0·52, *P* = 0·006). RVFW GLS correlated with mPAP (*R* = 0·68, *P*<0·001) and PVR (*R* = 0·59, *P* = 0·001). ROC curves for predicting PAH had AUC 0·73 for LV strain (*P* = 0·003) and 0·86 for RVFW GLS (*P*<0·001).

**Conclusions:**

Lower GLS is mainly determined by increased pulmonary pressure and not by SSc *per se*. Low LV and RVFW GLS are indicative of increased mPAP and PVR, which opens for improved non‐invasive methods to select patients eligible for right heart catheterization and to monitor the effects of PAH therapy.

## Introduction

Systemic sclerosis (SSc) is the connective tissue disease with the highest mortality rates, with more than half of the patients dying from disease‐related complications (Denton, 2015). Pulmonary arterial hypertension (PAH) accounts for up to 30% of mortality in SSc (Steen & Medsger, 2007). PAH is a rare and complex vascular disease (Peacock *et al*., 2007) with poor prognosis and high mortality rate, characterized by progressive increase in pulmonary vascular resistance (PVR) and loss of pulmonary vascular compliance (McLaughlin & McGoon, 2006; Galiè *et al*., 2016). Ultimately, these hemodynamic changes lead to right ventricular (RV) failure and hence rapid progression to death (Lai *et al*., 2014). Patients with SSc‐associated PAH (SSc‐PAH) have higher mortality than other types of PAH (Humbert *et al*., 2010; Thenappan *et al*., 2010; Benza *et al*., 2012; Lefèvre *et al*., 2013; Kylhammar *et al*., 2014; Korsholm *et al*., 2015).

Peak global longitudinal strain (GLS) by echocardiography has been shown to have prognostic value in SSc and PAH from various aetiologies (Goda *et al*., 2016; Cusmà Piccione *et al*., 2013; da Costa Junior *et al*., 2017; Henein *et al*., 2015). High negative GLS is a marker for good clinical outcome, while low negative GLS implies poor outcome (Goda *et al*., 2016; Cusmà Piccione *et al*., 2013; da Costa Junior *et al*., 2017; Henein *et al*., 2015). Using echocardiography and cardiac magnetic resonance (CMR), RV free wall (RVFW) GLS is usually low in patients with PAH and carries, in itself, prognostic value (Shehata *et al*., 2013; Goda *et al*., 2016; da Costa Junior *et al*., 2017). Furthermore, left ventricular (LV) GLS has been shown to be low in patients with PAH as well as in some patients with SSc, with higher risk of poor outcome (Spethmann *et al*., 2012; Cusmà Piccione *et al*., 2013; Hardegree *et al*., 2013; de Amorim Corrêa *et al*., 2016; Tennøe *et al*., 2018). However, some of these studies included SSc patients with PAH (Cusmà Piccione *et al*., 2013; Tennøe *et al*., 2018).The diagnostic and prognostic value of strain analysis with CMR in SSc with and without PAH has not yet been fully explored, and it is not known whether it is the pulmonary pressure or myocardial involvement of SSc, that is the major determinant for decreased strain.

Therefore, the aim for the present study was, by using CMR and feature tracking, to (i) evaluate whether LV and RVFW GLS differ between patients with SSc, with and without PAH, compared to healthy controls, and (ii) whether these differences are related to invasive measurements.

The outcome may be of great importance to differentiate SSc patients that exhibit PAH, enabling earlier referral for diagnostic right heart catheterization (RHC), and potentially identification at a lower risk status, rendering earlier treatment initiation and improved prognosis.

## Methods

### Study population

Consecutive patients with SSc examined with clinical indications for CMR at Skåne University Hospital during 2002–2015 were prospectively scanned and data retrospectively analysed. CMR data were analysed from 65 patients diagnosed with SSc, whereof 40 without PAH and 25 with PAH. Nineteen healthy controls, from earlier studies in our group (Steding *et al*., 2010; Bodetoft *et al*., 2011; Gyllenhammar *et al*., 2018), were matched with the patient population for gender and age (Mangion *et al*., 2016). Controls had no reported morbidities, including arterial hypertension, diabetes or coronary artery disease, no medical history or medication, and were checked by clinicians before entering the study.

The study was approved by the regional ethical committee of Lund, and subjects had given written informed consent prior to examination.

**Figure 1:**
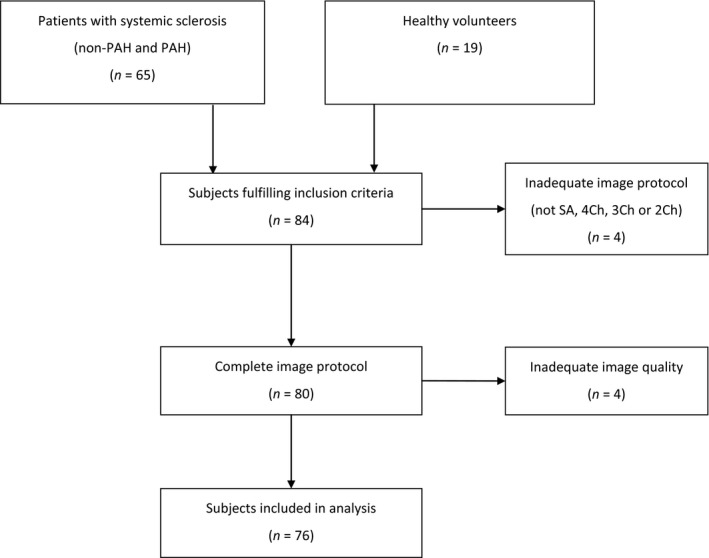
Flow chart for inclusion. Out of 84 subjects, eight were discarded as having inadequate image protocol or image artefacts. In the end, 76 subjects were used for the statistical analysis.

Patients were diagnosed with SSc at the Department of Rheumatology, Skåne University Hospital, Lund, fulfilling the American College of Rheumatology criteria for SSc (Masi *et al*., 1980). All patients were assessed for antibodies for SSc (anticentromere antibodies, anti‐RNA polymerase III antibodies, antitopoisomerase I antibodies/anti‐Scl‐70 antibodies and antinuclear antibodies) and for modified Rodnan skin score (mRSS). PAH was defined as mean pulmonary arterial pressure (mPAP) ≥25 mmHg and pulmonary arterial wedge pressure ≤15 mmHg at normal to low cardiac output measured with RHC (Galiè *et al*., 2016). RHC was performed as a part of the clinical evaluation in concordance with recommendations on detection of PAH in SSc patients (Coghlan *et al*., 2014). Clinical exclusion criteria for patients were postcapillary pulmonary hypertension, left‐sided heart failure, cardiac shunts and congenital heart disease. A technical exclusion flow chart is shown in Fig. 1. Fifty‐seven patients with SSc, 19 with and 38 without PAH and 19 healthy controls were used for calculations. One healthy control did not have RVFW GLS due to difficulties in strain analysis; however, all other parameters were successfully retrieved.

### Right heart catheterization

Right heart catheterization was performed at rest in the supine position with local anaesthesia, via an eight French sheath inserted in the right internal jugular vein using a triple‐lumen 7·3 French balloon‐tipped Swan–Ganz catheter. Pulsatile and mean right atrial pressures, pulmonary arterial pressures (PAPs) and pulmonary artery wedge pressures were recorded at free breathing over several heartbeats. Cardiac output was calculated via thermodilution, and PVR expressed as [PA_mean_ − PAwedge_mean_]/CO was computed. Systemic blood pressure was measured using a cuff and sphygmomanometer.

### Cardiac magnetic resonance

#### Image acquisition

**Figure 2:**
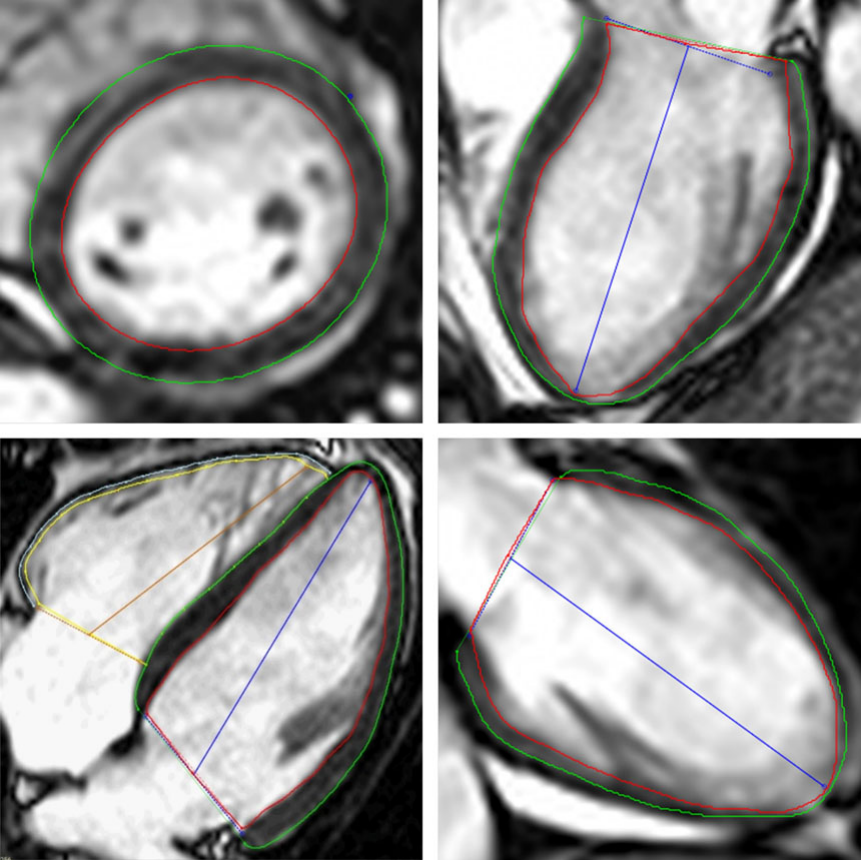
Example of delineations in one slice from a short‐axis stack (upper left), three‐chamber long‐axis view (upper right), four‐chamber long‐axis view (lower left) and two‐chamber long‐axis view (lower right). Red line represents left ventricle endocardial border, green line represents left ventricle epicardial border and blue lines represent left ventricle atrioventricular plane and ventricular axis to the apex. Yellow line represents right ventricle endocardial border, light blue line represents right ventricle epicardial border and the brown line represents the right ventricle atrioventricular plane and ventricular axis to the apex. [Color figure can be viewed at http://www.wileyonlinelibrary.com]

Cardiac magnetic resonance was performed on two 1·5‐Tesla MRI scanners (Philips Achieva, Best, the Netherlands and Siemens Aera, Erlangen, Germany). Short‐ and long‐axis cine steady‐state free precession images were acquired in supine position (Fig. 2). Typical image parameters were for Philips: temporal resolution of 47 ms reconstructed to 30 time phases per cardiac, 60° flip angle, 3 ms cycle repetition time, 1·4 ms echo time and slice thickness 8 mm with no slice gap; and for Siemens, temporal resolution was 46 ms reconstructed to 25 time phases per cardiac, 60° flip angle, 3 ms cycle repetition time, 1·4 ms echo time and slice thickness 6 mm with 2 mm slice gap.

Late gadolinium enhancement (LGE) was obtained on clinical indications to assess focal fibrosis. LGE images were acquired 10–15 min after intravenous gadolinium‐DTPA (Schering; 0·2 mmol kg^−1^) in short‐axis stack and long‐axis planes using an inversion‐recovery gradient echo sequence. Inversion times were adjusted to null the signal from normal myocardium. Severe renal failure with glomerular filtration rate below 30 mL min^−1^ 1·73 m^−2^ and allergic reaction to gadolinium was considered contraindications for contrast administration. Two patients with SSc‐PAH had no gadolinium administration due to impaired kidney function and two patients with SSc had termination of examination before gadolinium administration due to anxiety (*n* = 1) or claustrophobia (*n* = 1).

#### Image analysis

Image analysis and tissue tracking were performed with a CMR dedicated software for feature tracking (Circle CVI 42, Circle Cardiovascular Imaging Inc. Calgary, AB, Canada).

Endocardial and epicardial borders were manually delineated in end diastole and end systole in all short‐axis slices. End‐diastolic volume, end‐systolic volume, stroke volume, LV ejection fraction (EF) and cardiac output were calculated. Endocardial and epicardial borders were manually delineated in long‐axis two‐, three‐ and four‐chamber views in end diastole, and an automated propagation throughout the heart cycle was computed for each view (Fig. 2).

Endo‐ and epicardium of RVFW were manually delineated in the four‐chamber view, without including the septum, in end diastole and automatically propagated throughout the heart cycle (Fig. 2). Inadequate tracking for either the LV or the RV was manually corrected and recalculated. The presence of LGE was clinically assessed visually by experienced physician (E.O. >10 years of CMR experience).

All image analyses were performed blinded to clinical information by one observer (A.L.), with a second observer as adjudicator (E.O.) of the delineations.

#### Tissue tracking

In tissue tracking, a myocardial pattern is recognized by the software in the end‐diastolic image with the endocardium and epicardium manually delineated as external borders. The software propagates the delineations over the whole heart cycle.

Global longitudinal strain was defined as peak systolic strain. LV GLS was computed from the average of each segment according to a 17‐segment model from the long‐axis views (Cerqueira, 2002; Voigt *et al*., 2015) . RVFW GLS was calculated from the RVFW segments, excluding the septum. Low GLS is considered as a less negative value, and high GLS is a more negative value.

### Statistical analysis

Statistical analysis was performed using IBM SPSS Statistics 25, Armonk, NY, USA, IBM Corp and GraphPad Prism 7, La Jolla, CA, USA, GraphPad Software, Inc. Data are presented as mean ± standard deviation and 95% confidence interval. Group comparisons were done, for parametric data, with one‐way ANOVA and independent samples *t*‐test. For comparison between multiple groups, one‐way ANOVA in conjunction with Tukey *post hoc* analysis was used. Kruskal–Wallis test was used for non‐parametric data. Two‐sided chi‐square was used for nominal data. Linear regression was used for correlation analyses. Multivariate linear regression was used for assessing the added value of RVFW GLS to RVEF for correlation with mPAP and PVR. For cut‐off value calculation, a sum of squares method was used (Froud & Abel, 2014). Statistical significance was assumed when a two‐sided *P*‐value was <0·05.

## Results

Characteristics for patients and controls are shown in Table 1, and antibody profiles are shown in Table 2.

**Table 1:** Patient characteristics

	Diagnosis			*P*‐value		
	Control (*n* = 19)	SSc (*n* = 38)	SSc‐PAH (*n* = 19)	*P*‐value^b^	*P*‐value^c^	*P*‐value^d^
Gender; Female	68%	87%	68%	0·1	1·0	0·1
Age	59 ± 15	57 ± 11	64 ± 13	0·8	0·4	0·1
BSA (m^2^)	1·8 ± 0·2	1·8 ± 0·2	1·8 ± 0·2	0·7	1·0	0·9
NIBPs (mmHg)	128 ± 12^a^	130 ± 22	123 ± 18	0·9	0·7	0·4
NIBPd (mmHg)	76 ± 9^a^	74 ± 9	73 ± 11	0·8	0·7	1
CMR						
LVEF (%)	58 ± 5	62 ± 6	50 ± 9	0·1	0·001	<0·001
HR (/min)	62 ± 8	74 ± 9	87 ± 13	<0·001	<0·001	<0·001
LVEDVI (mL m^−2^)	87 ± 14	75 ± 14	64 ± 13	0·005	<0·001	0·02
LVESVI (mL m^−2^)	37 ± 8	29 ±8	32 ± 9	0·004	0·2	0·3
LVSVI (mL m^−2^)	50 ± 8	45 ± 9	31 ± 8	0·08	<0·001	<0·001
CI (L min^−1^ m^−2^)	3·1 ± 0·6	3·3 ± 0·7	2·7 ± 0·7	0·5	0·2	0·006
RVEF (%)	57 ± 5	60 ± 7	38 ± 10	0·2	<0·001	<0·001
RVEDVI (mL m^−2^)	91 ± 20	73 ± 13	100 ± 28	0·02	0·4	<0·001
RVESVI (mL m^−2^)	40 ± 11	29 ± 9	63 ± 24	0·03	<0·001	<0·001
RVSVI (mL m^−2^)	52 ± 11	44 ± 6	37 ± 9	0·002	<0·001	0·03
RHC		*N* = 9	*N* = 18			
sPAP (mmHg)		31 ± 7	65 ± 19			<0·001
mPAP (mmHg)		18 ± 3	39 ± 11			<0·001
PAWP (mmHg)		7 ± 4	5 ± 3			0·1
mRAP (mmHg)		2 ± 1	5 ± 5			0·1
PVR (wood units)		2 ± 1	7 ± 4			<0·001
CI (L min^−1^ m^−2^)		3·1 ± 0·6	3·1 ± 0·8			0.9
Comorbidities						
Smoker; yes/ex		13/29%	5/53%			0·2
Diabetes; yes		13%	16%			0·8
Raynaud's		100%	100%			1
Limited/diffuse SSc		79/21%	95/3%			0·1
mRSS		5 ± 9	1 ± 2			0·03
IHD		5%	8%			0·7
SSc duration		4·3 ± 6·8	4·8 ± 5·0			0·8
ACE/ARB		11%	21%			0·3
CCB		39%	47%			0·6
BB		5·3%	5·3%			1
Statin		2·6%	11%			0·2
ERA		0%	63%			<0·001
PDEI5		0%	26%			0·001
Prostanoid		0%	5·3%			0·2
NSAID		5·3%	16%			0·2
Corticosteroid		13%	21%			0·4
Immunosuppressant		7·9%	16%			0·4

Control, healthy adult volunteers; SSc‐PAH, systemic sclerosis with pulmonary arterial hypertension; SSc, systemic sclerosis without pulmonary arterial hypertension; BSA, body surface area; NIBPs, non‐invasive blood pressure, systolic; NIBPd, non‐invasive blood pressure, diastolic; CMR, cardiac magnetic resonance imaging; LVEF, left ventricle ejection fraction; HR, heart rate; LVEDVI, left ventricle end‐diastolic volume index to BSA; LVESVI, left ventricle end‐systolic volume index; LVSVI, left ventricle stroke volume index; RVEDVI, right ventricle end‐diastolic volume index; RVESVI, right ventricle end‐systolic volume index; RVSVI, right ventricle stroke volume index; CI, cardiac index; RHC, right heart catheterization; sPAP, systolic pulmonary artery pressure; mPAP, mean pulmonary artery pressure; PAWP, pulmonary artery wedge pressure; mRAP, mean right atrial pressure; PVR, pulmonary vascular resistance; mRSS, modified Rodnan skin score; IHD, ischaemic heart disease; SSc duration in years; ACE/ARB, Ace inhibitor/angiotensin II blocker; CCB, dihydropyridine calcium channel blocker; BB, beta blocker; ERA, endothelin receptor antagonists; PDEI5, phosphodiesterase type 5 inhibitor; NSAID, non steroidal anti‐inflammatory drugs.

a *n* = 17.

b *P*‐value: comparison between control and SSc.

c *P*‐value: comparison between control and SSc‐PAH.

d *P*‐value: comparison between SSc and SSc‐PAH.

**Table 2:** Antibodies for systemic sclerosis

	ACA	ARA	ATA	ANA+	ANA−
SSc‐PAH	7 (37%)	0	1 (5%)	11 (58%)	0
SSc	16 (42%)	4 (11%)	5 (13%)	10 (26%)	3 (8%)

Values expressed in absolute numbers and percentage in parenthesis. SSc‐PAH, systemic sclerosis with pulmonary arterial hypertension; SSc, systemic sclerosis without pulmonary arterial hypertension; ACA, anti centromere antibodies; ARA, anti RNA polymerase III antibodies; ATA, anti topoisomerase I antibodies (anti‐Scl‐70 antibodies); ANA, anti nuclear antibodies (without ACA, ARA or ATA). *P* = 0·1 between SSc‐PAH and SSc for all antibodies.

**Figure 3:**
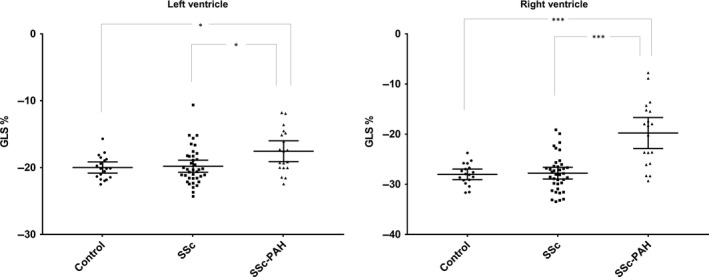
Peak global longitudinal strain (GLS) in controls, systemic sclerosis (SSc) and systemic sclerosis with pulmonary arterial hypertension (SSc‐PAH) in mean ± CI (95%). Left panel shows peak left ventricular (LV) GLS. Right panel shows GLS in the right ventricular (RV) free wall. Control group (dots) (LV:* n* = 19, RV:* n* = 18), patients with SSc (squares) (*n* = 38) and patients with SSc‐PAH (triangles) (*n* = 19). *P<0·05, ***P<0·001

Peak global longitudinal LV strain was lower in SSc‐PAH (−18 ± 3%) compared to both SSc (−20 ± 3%, *P* = 0·01) and controls (−20 ± 2%, *P* = 0·02). LV strain did not differ between SSc and controls (*P* = 1) (Fig. 3).

Peak longitudinal RVFW strain was lower in SSc‐PAH (−20 ± 6%) compared to both SSc (−28 ± 4%, *P*<0·001) and controls (−28 ± 2%, *P*<0·001). RVFW strain did not differ between SSc and controls (*P* = 1·0) (Fig. 3).

### Correlations between longitudinal strain and invasive measures

**Figure 4:**
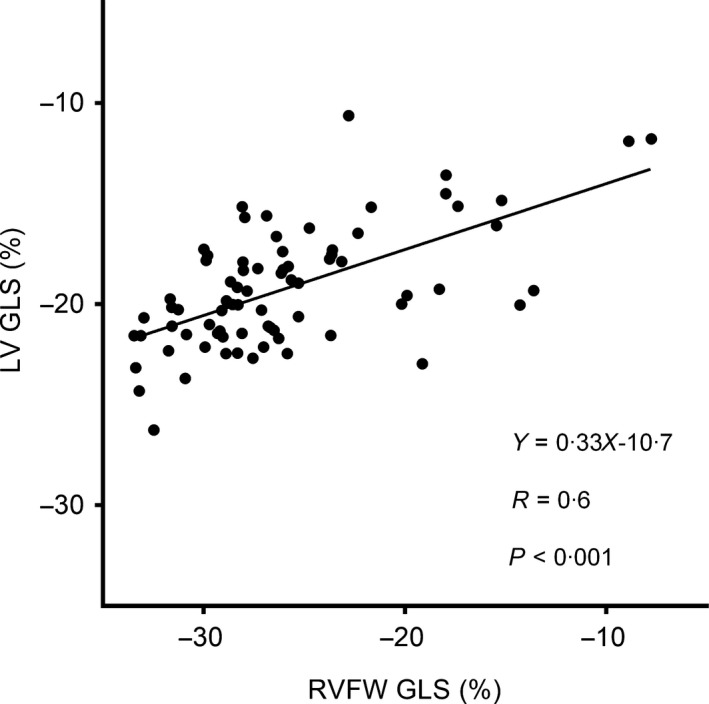
Linear regression analysis between peak right ventricular free wall global longitudinal strain (RVFW GLS) and peak left ventricular global longitudinal strain (LV GLS).

**Figure 5:**
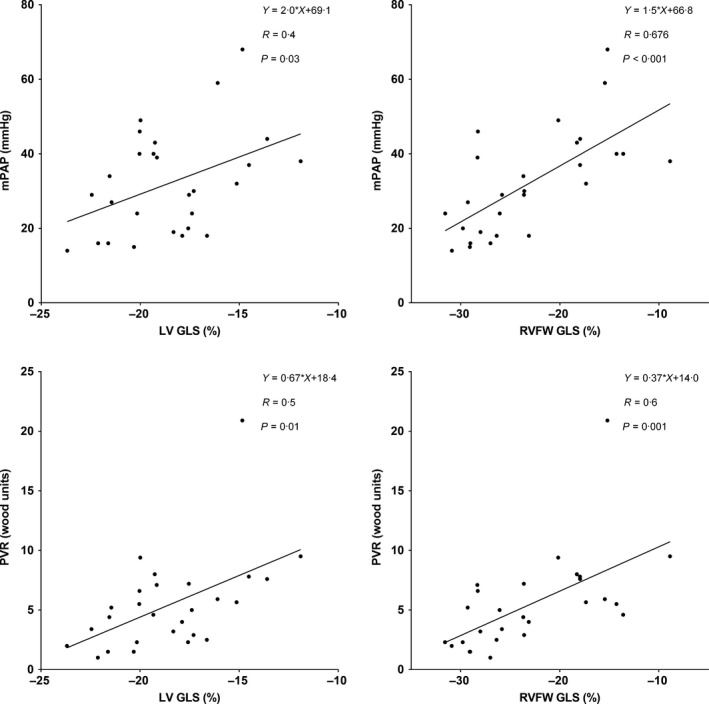
Linear regression analyses between peak global longitudinal strain (GLS) and mean pulmonary arterial pressure (mPAP, top) and pulmonary vascular resistance (bottom). Left ventricular LV GLS (left panels) and right ventricular free wall (RVFW) GLS (right panels).

Peak RVFW strain correlated with LV strain (*R* = 0·62, *P*<0·001) (Fig. 4). RVFW strain correlated with mPAP (*R* = 0·68, *P*<0·001) (Fig. 5) and with PVR (*R* = 0·59, *P* = 0·001) (Fig. 5). RVFW strain correlated with both systolic PAP (*R* = 0·65, *P*<0·001) and diastolic PAP (*R* = 0·76, *P*<0·001). LV strain correlated with mPAP (*R* = 0·42, *P* = 0·03) and PVR (*R* = 0·52, *P* = 0·006) (Fig. 5). RVEF correlated with mPAP (*R* = 0·69, *P*<0·001). Multivariable linear regression with RVFW strain and RVEF correlated with mPAP (*R* = 0·76, *P*<0·001) and with PVR (*R* = 0·65, *P* = 0·001).

**Figure 6:**
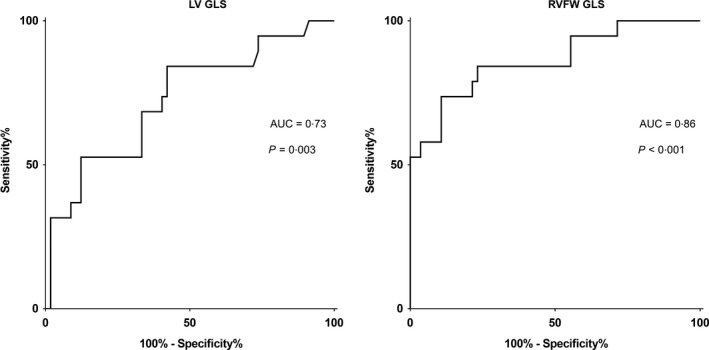
Receiver operating characteristics analyses for predicting pulmonary arterial hypertension with peak global longitudinal strain (GLS). Left panel shows peak left ventricular (LV) GLS. Right panel shows GLS in the right ventricular free wall (RVFW). AUC, area under the curve.

Analysis of receiver operating characteristics for RVFW strain predicting the diagnosis of PAH, using mPAP ≥25 mmHg from RHC as reference, showed an area under the curve of 0·86 (*P*<0·001). A cut‐off value of RVFW strain at −26·2% gave a sensitivity of 84% and a specificity of 77% for the diagnosis of PAH (Fig. 6). For LV strain predicting PAH, area under the curve was 0·73 (*P* = 0·003) and a cut‐off value of LV strain at −20·0% gave a sensitivity 84% and specificity 58% (Fig. 6). Area under the curve for RVEF predicting the diagnosis of PAH was 0·96 (*P*<0·001), and a cut‐off of 52·0% gave a sensitivity of 95% and a specificity of 84%. Area under the curved for combined RVFW strain and RVEF was 0·98 with a sensitivity of 90% and a specificity of 90%.

### Strain and fibrosis

Four patients had manifestations of infarction, 16 had focal fibrosis at RV insertion and 33 patients were without visually localized fibrosis and scar. LV strain was lower in patients with insertion fibrosis (−18 ± 3%) and even lower in patients with infarction (−17 ± 4%) compared to those without fibrosis (−20 ± 2%, *P* = 0·005 among the groups). RVFW strain was lower in patients with fibrosis (−22 ± 8%) and in patients with infarction (−22 ± 5%) compared to patients without fibrosis (−27 ± 4%, *P* = 0·007 among the groups). Both LVEF and RVEF were lower in patients with fibrosis or infarction compared to patients without fibrosis (*P* = 0·004 and *P*<0·001).

## Discussion

Our study suggests that lower longitudinal strain in the SSc‐PAH patients is associated with PAH rather than SSc *per se*. Left and right ventricular strain in SSc did not differ from control, whereas strain in SSc‐PAH did. On the other hand, both LV and RVFW GLS decreased with increased mPAP and PVR in the whole patient population.

The lower LV strain in the SSc‐PAH patients is in concordance with the study by Corrêa *et al*. that showed a lower longitudinal strain in PAH patients compared to controls (de Amorim Corrêa *et al*., 2016). However, in that study, mostly patients without SSc were included and SSc‐PAH might not be phenotypically representative for the whole group of PAH (Chung *et al*., 2010; Hsu *et al*., 2011). The decline in LV function due to PAH is in concordance with a study by Hardegree *et al*. (2013) using echocardiography and with an earlier study using CMR showing altered regional contribution to ventricular stroke volume in pulmonary hypertension (Ostenfeld *et al*., 2016).

In our study, we did not find a lower LV longitudinal strain in the patients with SSc compared to healthy controls. This is in contrast to earlier studies that have shown that LV longitudinal strain is lower in patients with SSc compared to controls using echocardiography (Spethmann *et al*., 2012; Cusmà Piccione *et al*., 2013; Tennøe *et al*., 2018). In the echocardiographic studies by Spethmann *et al*. and Tennøe *et al*., LV GLS was in proximity (SSc −19% versus controls −21%, *P*<0·008 and SSc −18% versus controls −20%, *P*<0·05) to our LV values (GLS −20% for both groups). However, in the study by Piccione *et al*., SSc patients had much lower LV GLS compared to controls (−13% versus −23%, *P*<0·001) (Cusmà Piccione *et al*., 2013). In the study by Tennøe *et al*., 88 out of 333 patients had PAH, and in the study by Piccione *et al*., patients with estimated elevated PAP from echocardiography, suggesting the presence of PAH, were included in the SSc group (Cusmà Piccione *et al*., 2013; Tennøe *et al*., 2018). The low strain in these studies could be derived from the inmixing of patients with SSc and PAH and might therefore not be in conflict with our results. More than half of the patients in the study by Piccione *et al*. had anti‐Scl‐70 antibodies (ATA), indicating that their patients were more severely ill at least from a fibrotic point of view, and they found a correlation between GLS and anti‐Scl‐70 antibodies (Cusmà Piccione *et al*., 2013). In our study, a minority of patients had anti‐Scl‐70 antibodies (ATA, Table 2). Nonetheless, antibodies did not show any difference between SSc and SSc‐PAH in this study. Patients in our study are likely not affected by a particularly severe form of disease, as confirmed by antibodies with a low number of patients with ATA. It should also be noted that the studies by Spethmann, Tennöe and Piccione were performed with echocardiography. Speckle tracking by echocardiography and feature tracking by CMR are not directly interchangeable, and a previous study correlating LV GLS from CMR feature tracking and echocardiographic speckle tracking showed *R* values between 0·83 and 0·87 (Obokata *et al*., 2016). Of note, in the latter study, the same vendor was used for CMR and echocardiography analysis. There is a lack of studies investigating the intervendor variability of MR feature tracking. Notably, intervendor differences in strain values among vendor specific echocardiographic machines and software have been presented (Farsalinos *et al*., 2015; Mirea *et al*., 2018). This, in addition to intermodality variability, makes it difficult to compare absolute values and to our knowledge there are no normal values, with regard to gender and age, for the specific software used. Thus, methodological differences in speckle tracking and feature tracking might explain different findings in our study compared to the echocardiographic studies with regard to LV GLS (Spethmann *et al*., 2012; Cusmà Piccione *et al*., 2013; Tennøe *et al*., 2018).

As expected, the SSc‐PAH group had significantly lower peak RVFW GLS compared to controls. This is in agreement with earlier studies showing that RVFW GLS is lower in patients with PAH using echocardiography (de Amorim Corrêa *et al*., 2016; Goda *et al*., 2016). Furthermore, Goda *et al*. (2016) have shown that lower RVFW GLS leads to worse prognosis. On the other hand, the SSc patients did not show any tendencies for lower RVFW GLS in our study.

A regression analysis performed on the subjects in our study shows a correlation between LV and RVFW GLS (Fig. 5). An impaired LV regional function has earlier been suggested related to impaired RV function due to pressure‐loaded RV and impaired LV filling (Marcus *et al*., 2001; Gurudevan *et al*., 2007). Since patients with SSc‐PAH have both lower LV and RVFW GLS, it could be assumed that there is a cause–effect relation between lower RV strain and lower LV strain. In other words, a decreased RV longitudinal function implies a decreased LV regional function due to LV underfilling despite preserved LV EF (Marcus *et al*., 2001).

We found correlations between LV and RVFW GLS and both mPAP and PVR with the strongest correlation between RVFW GLS and mPAP. Similar correlations with RVFW GLS have earlier been found by Shehata *et al*. (2013). These correlations are of interest, since it has been suggested that RVFW GLS could be a method for non‐invasive evaluation of PAP in a study by Shiino *et al*. (2015) using echocardiography in patients with chronic thromboembolic pulmonary hypertension. Our study supports that RVFW GLS with CMR can be an indicator of elevated pressure in the pulmonary circulation. However, RVEF was significantly reduced in SSc patients with PAH compared to SSc patients without PAH and in controls. RVEF was also shown to have a good correlation with mPAP and PVR and multivariate linear regression combining RVFW GLS and RVEF showed a higher correlation than with either RVEF or RVFW GLS alone. This suggests that RVFW strain has an added value to RVEF and helps predict PAH in SSc patients. Furthermore, it appears that the impaired LV GLS is partly due to increased pressure and resistance in the pulmonary circulation, which could be due to a true myocardial dysfunction but could also imply underfilling of the LV. LV and RVFW GLS were lower in patients with both insertion fibrosis and infarction compared to patients without fibrosis. However, the sample of patients with infarction was very low with only four patients. Freed *et al*. (2012) have showed that insertion fibrosis with LGE is an indicator of poor prognosis.

Left ventricular and RVFW GLS were shown to have diagnostic value for predicting PAH in our study, with RVFW showing a larger area under the curve and a higher sensitivity of the two. These findings are in concordance with Shiino *et al*. (2015). RV EF was also shown to have diagnostic value for predicting PAH with a larger area under the curve compared to RVFW GLS. Multivariate analysis, combining RV EF and RVFW GLS, had an even greater area under the curve for predicting PAH.

The values of the mRSS indicates disease stage. MRSS is shown in Table 1 and indicates a later stage of disease in patients with SSc‐PAH compared to SSc group; however, we found no difference in mean SSc duration between patients with and without PAH. It has been reported that the mRSS is worsened in the early disease stage and declines in the late disease, why mRSS has been used as a surrogate measure of SSc severity, and, furthermore, is associated with involvement of internal organs including heart and lung (Amjadi *et al*., 2009). These findings could explain the reduction in GLS in SSc‐PAH compared to SSc group on the basis of more myocardial affection beside the lung affection in these patients. Lower GLS may be related to PAH, but a relation between lower GLS and myocardial fibrosis cannot be excluded in SSc, depending on disease's severity and extent.

Difference in afterload and heart rate could affect the strain values. However, controls as well as SSc patients with and without PAH had equal blood pressures. Also, higher heart rate reduces diastolic filling time and could decrease atrial filling and thereby the filling of the ventricles. The heart rate was higher in SSc patient than controls which is in concordance with a study by Bourji *et al*. (2017). In our study, however, there was no difference in strain between these two groups. On the other hand, the PAH group had the highest heart rate in parity with the levels shown by Bourji *et al*. which could alter the filling of the ventricles and thereby resulting in lower strain on both sides.

Tissue tracking can be performed without contrast or special protocols and can be added to existing CMR analysis. This could be a field where CMR could have a significant use as a complement to RHC in screening, diagnosis and follow‐up of SSc‐PAH patients. Even if echocardiography is the first‐line non‐invasive modality in assessing PAH, the method is challenged in the assessment of the right ventricle (Ostenfeld & Flachskampf, 2015). This is an area where strain with CMR could provide incremental value.

### Limitations

Radial or circumferential strain was not assessed in this study. LV and RVFW GLS were chosen according to recommendations (Voigt *et al*., 2015). It has been shown that LV GLS has a higher reproducibility than LV radial and circumferential strain and that RVFW GLS has been shown to have prognostic value in various diseases using echocardiography (Yingchoncharoen *et al*., 2013; Lang *et al*., 2015).

A study by Stamm *et al*. (2016) has shown that exercise pulmonary haemodynamics can be useful for diagnosis and prognosis of patients with SSc with regard to PAH. However, exercise indices have been removed from the current guidelines on PAH and were not in the scope of this study (Badesch *et al*., 2009). Future studies on strain and exercise in SSc and PAH would be of interest.

Healthy controls and some SSc patients did not have data from RHC, since RHC was only done on clinical indication and in concordance with evidence‐based detection of PAH in SSc (Coghlan *et al*., 2014). One patient with PAH had no RHC due to comorbidities contraindicating invasive measurements, although all other clinical indications, including echocardiography, concluded the diagnosis. It would have been preferable to have invasive measures in all patients. On the other hand, a large proportion of patients had invasive measures and SSc patients followed the screening algorithm for detecting PAH, why this might not be considered a major limitation. We still found positive correlations between GLS, mPAP and PVR.

Another confounder to the strain values could be medical therapy, where patients receiving therapy could have increased longitudinal strain. However, the only differences in cardiac medication between the groups were higher usage of endothelin receptor antagonists and phosphodiesterase type 5 inhibitor in the PAH group (Table 1). There were less PAH patients being treatment naive at CMR examination compared to patients without PAH (2 versus 18, *P*<0·006). This would suggest that treatment naivety is not the cause of lower strain values.

## Conclusion

A decrease in LV and RVFW GLS is more likely associated with PAH than SSc *per se*. The correlation between left and right GLS and PAPs could open possibilities for non‐invasive evaluations of PAPs in patients with early signs of SSc‐PAH and to select patients eligible for RHC and monitor effects of PAH therapy. This is of importance as earlier diagnosis of PAH, at a lower risk status, previously has been shown to be related to an improved survival (Kylhammar *et al*., 2017).

## Conflict of interest

Outside the submitted work, H.A. is a shareholder in Imacor AB, Lund, Sweden.
